# Supplementary material for: Sphingolipid Profile during Cotton Fiber Growth Revealed That a Phytoceramide Containing Hydroxylated and Saturated VLCFA Is Important for Fiber Cell Elongation
Source: Biomolecules. 2021 Sep 12;11(9):1352. doi: 10.3390/biom11091352 (PMC8466704; doi:10.3390/biom11091352)
Supplement: Supplementary file 1 [file biomolecules-11-01352-s001.zip › biomolecules-1362178-final-suppl/Supplementary File/Table S1.pdf]

Supplementary Information

Table S1. Gene-specific primers used in RT-PCR analysis.

Gene Name	Primers (5′→ 3′)
GhHis-F	CCGTCCTGGAACTGTTGCTCT
GhHis-R	ACCCACAAGGTATGCCTCTGC
GhSPT1-RT-F	CTCGCTCTCGATAAATGCCCCC
GhSPT1-RT-R	CACATGCCATAAGCAGGTCGGA
GhSPT2-RT-F	TACAGTTGCTTTCCCTGCCACC
GhSPT2-RT-R	TGATTGTGTCTTTCCCCGGCTG
GhKSR1-RT-F	AGACAAGGGACCAGCCTCCATT
GhKSR1-RT-R	AATCCCGGAGTTTCAGTCACGG
GhKSR2-RT-F	TAGAAACAAACTCAAACCTGG
GhKSR2-RT-R	CTCAAACCTGGTTCAAATCC
GhSBH1-RT-F	AAGACCGTCATCAAAGGCGTCC
GhSBH1-RT-R	CCTGAAGAAGCTTCGGCATCGT
GhSBH2-RT-F	CCGGCTGGTTGTTCCCTATT
GhSBH2-RT-R	TGGACATCATGGTATGCCGA
GhSBH3-RT-F	GGTTCCCTACGCATTTGGAGCA
GhSBH3-RT-R	CCCGCAATGGTCATCCACTGAT
GhLOH2-RT--F	TCGATTTCCGTGGACCAAGAGC
GhLOH2-RT--R	GCTGCTGATGTTGTTACGTCGC
GhLOH3-RT-F	GCTGAAACACTTGCTAGCTTCGC
GhLOH3-RT-R	TAGCAAATTGGCCCATCCACTG
Gh4DES1-RT-F	AGGTGAAAGAAATCGCACCCGA
Gh4DES1-RT-R	CGGTCCGACGGTTTGATCCATT
Gh8DES1-RT-F	GCCAAGGTGCCATCTCAGGAAA
Gh8DES1-RT-R	TGGGTTGCTGAGAATCCTTGCC

Gh8DES2-RT-F	GCTGTGACCGGTATCCAACA
Gh8DES2-RT-R	AATGGCACCTAGGCAACCTC
GhGCS1-RT-F	ATGACCGGAGATGGTCGAAGGA
GhGCS1-RT-R	GGGAGCTGGAAATGGAGTGACG
GhACER3-1-RT-F	CTCGCGAAGTTGTACGTGGCTA
GhACER3-1-RT-R	ATGACCTTGAGGGTTGATCGGC
GhACER3-2-RT-F	CTCGCGAAGTTGTACGTGGCTA
GhACER3-2-RT-R	ATGACCTTGGGGGTTGATTCGC
GhNCER2-RT-F	GCTTGCTACATCCTTCCGCTCA
GhNCER2-RT-R	AGCTCATTCTTTCCACCGCAGG
GhFAH1-RT-F	GCCTGTTGTTTGCTGGTGCATT
GhFAH1-RT-R	TTGTGGTGGCAGCCATGAAGAA
GhKCS1-RT-F	AAAGCATTGATGCCGCAACGAG
GhKCS1-RT-R	CTCCGTGAAAGCACCGCTATCT
GhKCS2-RT-F	GAACAAGGCACCATTGGCATCG
GhKCS2-RT-R	GAGAACGAGTGGGGCAAGTGTT
GhECR-RT-F	GGAGAGGCCTGTCGTCCTTAAC
GhECR-RT-R	GAACAGGGTGGATGACACGCTT
GhHCD-RT-F	TGCTGTCGAAAAGCCTCTCCTT
GhHCD-RT-R	TGACACCGGTGACCTAACCAGA
GhKCR1-RT-F	TCGTGGCCACCAAAATGACGAA
GhKCR1-RT-R	CTCATCATGGCCAACCCATCGT
